# Effective hybrid search technique based constraint mixed-integer programming for smart home residential load scheduling

**DOI:** 10.1038/s41598-023-48717-x

**Published:** 2023-12-10

**Authors:** Esam H. Abdelhameed, Samah Abdelraheem, Yehia Sayed Mohamed, Ahmed A. Zaki Diab

**Affiliations:** 1 Faculty of Energy Engineering, Aswan University, Aswan, Egypt; 2 Modern University for Technology and Information, Cairo, Egypt; 3 Faculty of Engineering, Minia University, Minia, Egypt

**Keywords:** Electrical and electronic engineering, Energy science and technology

## Abstract

In this paper, the problem of scheduling smart homes (SHs) residential loads is considered aiming to minimize electricity bills and enhance the user comfort. The problem is addressed as a multi-objective constraint mixed-integer optimization problem (CP-MIP) to model the constrained load operation. As the CP-MIP optimization problem is non-convex, a novel hybrid search technique, that combines the Relaxation and Rounding (RnR) approach and metaheuristic algorithms to enhance the accuracy and relevance of decision variables, is proposed. This search technique is implemented through two stages: the relaxation stage in which a metaheuristic technique is applied to get the optimal rational solution of the problem. Whereas, the second stage is the rounding process which is applied via stochastic rounding approach to provide a good-enough feasible solution. The scheduling process has been done under time-of-use (ToU) dynamic electricity pricing scheme and two powering modes (i.e., powering from the main grid only or powering from a grid-tied photovoltaic (PV) residential power system), in addition, four metaheuristics [i.e., Binary Particle Swarm Optimization (BPSO), Self-Organizing Hierarchical PSO (SOH-PSO), JAYA algorithm, and Comprehensive Learning JAYA algorithm (CL-JAYA)] have been utilized. The results reported in this study verify the effectiveness of the proposed technique. In the 1st powering mode, the electricity bill reduction reaches 19.4% and 20.0% when applying the modified metaheuristics, i.e. SOH-PSO and CL-JAYA, respectively, while reaches 56.1%, and 54.7% respectively in the 2nd powering scenario. In addition, CL-JAYA superiority is also observed with regard to the user comfort.

## Introduction

Energy is one of the worthy resources in everyday life. However, human consumption of energy, i.e., energy demand, increases gradually over time. In 2020, global residential building’s operations accounts for 22 percent of global energy consumption^1,2^, which is considered a large part of the overall percentage of energy consumption. High energy consumption of residential buildings has critical consequences on the economy and grid reliability. In this aspect, more enhanced management methodologies should be adopted to overcome such challenge. In the literature this problem has been handled via integrating Demand Side Management (DSM) systems with the traditional grids to transfer the traditional power networks into smart ones. This transition is combined with integrating Renewable Energy Sources (RES) into the grid, leading to offer number of advantages like increased efficiency and sustainability of the grid^3^. Recently, Smart home (SH) technologies have been presented as a promising solution to conventional power system problems. Smart homes with appropriate sizing and efficient energy management systems have the ability to solve such problems, leading to more efficient, reliable, sustainable and low carbon energy infrastructures. Further, due to their features, i.e., home automation, security, energy efficiency, productivity enhancement, user comfort enhancement, and energy savings, SHs technologies have a potential spread in the upcoming future^4,5^.

It is noticed from the literature that, for managing the energy consumption in the smart grids, applicable dynamic pricing schemes were introduced instead of the flat rate pricing schemes. These electricity tariffs offer different time-based pricing rates e.g. Time-of-Use (ToU) pricing, Real-Time Pricing (RTP), Critical Peak Pricing (CPP), and Day-Ahead Pricing (DAP)^6–9^. In ToU pricing scheme, the price rate varies related to the time in the day, whereas, the rate in DAP pricing is provided by utilities depending on deals for setting supply–demand balance in hourly intervals. However, the price rate in RTP scheme is the rate of real electric power delivery varying from hour to hour.

The demand side management is considered a key technology in the SHs and smart grids for providing economic electricity bills, and enhancing the user comfort^10^. Energy demands can be managed efficiently by the DSM through sharing the allowed real-time information between the consumer, residential power system, and the utility grid, which helps in keeping the supply and demand of electricity in balance with consideration of the dynamic electricity tariff and user comfort. Through DSM adaptation, the customers have the ability to reduce their energy consumption during on-peak periods and shift it to the non-peak periods in response to dynamic pricing rates, which is called demand response^11–13^. The demand response program has two major features: the first one arises from utility viewpoint as providing the Peak-to-Average Ratio (PAR) minimization capability resulting in more stability of the grid, while the second feature is allowing the consumers to consume their high-peak energy during low-peak hours of the grid leading to low electricity bills^13,14^. In the literature, many recent works have been conducted on DSM and SHs technologies discussing how to keep control on electricity consumption through optimal design and programming. All these studies have common objectives of minimizing costs, reducing PAR, maximizing customer comfort, reducing carbon emissions (based on RES integration into the smart grid). Whereas several solutions were introduced to address the SHs' appliance scheduling problem based on metaheuristic optimization techniques. In^13,14^, the authors suggested efficient integration of RESs with battery storage systems for solving the energy management issue, and reducing electricity bills, PAR and carbon emissions. The obtained load scheduler and controllers of the energy storage system management were designed depending on metaheuristic techniques i.e., the genetic algorithm (GA), wind driven optimization (WDO), BPSO, bacterial foraging algorithm (BFA), and hybrid approach of GA, WDO, and PSO. In^15^, for getting the DSM objectives, the authors introduced optimal home energy management controllers. The study was done on a single and multiple homes under the RTP and Critical Peak Pricing (CPP) schemes. The authors designed the controller based on constrained optimization utilizing heuristic algorithms i.e., GA, WDO, harmony search algorithm (HSA), and the genetic harmony search algorithm which enhanced the search efficiency and dynamic capability to obtain better solutions comparing to the other techniques. The authors in^16^ applied a Home Energy Management System (HEMS) based on a pricing-based demand response considering user satisfaction. The authors incorporated various household appliances; in addition they involved energy storage system and distributed energy resources. Further, they developed an adequate energy consumption model for different SHs' appliances; the introduced HEMS offered solutions with various user satisfaction levels. In^18^, BFA and PSO-based energy management system were utilized in the DSM framework to accomplish its objectives. Sattarpour et al.^19^ suggested multiple integration linear programming approaches for residential loads scheduling. The main objectives of the study were the cost reduction and load curve linearization, however, user comfort was not taken into consideration. The authors of^20^ suggested GA to implement optimal DSM strategies for SHs' energy demand management. The authors discussed the operational power of household appliances; however no RESs were considered in this study. The obtained results were compared to those of WDO, GA outperforms the WDO in terms of energy cost and PAR reduction which are reduced by 29.0% and 36.2%, respectively. In^21^, a smart load management system based on evolutionary techniques was suggested to schedule the energy of a residential SH in response to a Time-of-Use (ToU) dynamic pricing rate. The authors introduced a model for the smart HEMS where the evolutionary techniques were implemented to solve the scheduling problem for two different operating scenarios: the 1st is supplying the SH from the utility grid only whereas the 2nd scenario depends on integrating a photovoltaic (PV) system to the residential microgrid. In^22^, a programmable energy management controller based on heuristic approaches was presented to simultaneously regulate energy consumption in residential buildings for reducing PARs, carbon emissions, and user discomfort. The proposed building model depends on PV panels as a renewable energy source. The authors suggested different heuristic optimization techniques for the controller design such as GA, BPSO, ACO, WDO, BFA, and hybrid GA-PSO. In order to manage the power usage of IoT-enabled residential building smart appliances, reduce electricity bill, and enhance user comfort, the authors of^23^ suggested a wind-driven bacterial foraging scheme, which is a combination of two algorithms i.e., wind-driven optimization (WDO) and bacterial foraging optimization (BFO).

Motivated by the aforementioned literature works and aiming for home automation, economic electricity bills and user comfort, this study targets designing an optimized load scheduler for smart homes. The designed systems have been simulated under ToU dynamic pricing scheme. Besides, a PV residential power system has been integrated to the SHs' powering network to reduce the dependability on the utility grid and confirm the effectiveness of renewable resources utilization on achieving the research objectives. The main contributions of this research can be summed up as follows:

1. For reducing the electricity cost while enhancing the consumers comfort, this paper studies the employment of combined mixed-integer programming with constraint programming (CP-MIP) on the problem of smart home residential loads scheduling to optimally estimate a day-ahead scheduling of the residential loads while modeling the constraints of the load operation.
2. As the CP-MIP is a non-convex optimization problem, a novel hybrid search technique has been employed. This technique combines Relaxation and Rounding (RnR) approach and metaheuristic algorithms and is implemented through two stages: the relaxation stage, in which a metaheuristic algorithm is applied to get the optimal rational solution of the problem, whereas, the second stage is the rounding process which is applied via stochastic rounding approach to provide a good-enough feasible solution of the scheduling problem.
3. Two modified metaheuristics approaches are applied on the relaxation and rounding scheme i.e., Self-Organizing Hierarchical PSO (SOH-PSO) and Comprehensive Learning JAYA algorithm (CL-JAYA), while the obtained results are compared with those of the original algorithms i.e., the Binary Particle Swarm Optimization (BPSO), and the JAYA algorithm.
4. Incorporating plug-in electric vehicles (EVs) as electrical loads of future smart homes with real-time charging profiles. The EVs are modeled as fixed and interruptible loads for fast and normal charging, respectively.
5. The proposed approach is appealing not just for smart homes load scheduling problem, but for any mixed-integer real-world applications.

The rest of this article is organized as follows: Section “Architecture of the proposed smart HEMS” presents a study on the smart home technology and the architecture of the proposed smart HEMS including modeling of the residential Loads, EVs, PV system, user comfort, and the pricing rates. The design of smart scheduler including the problem formulation and the proposed solution is described in Section “Formulation of load scheduling problem”, whereas in Section “Proposed smart load scheduling design methodology”, the proposed relaxation and rounding technique for smart scheduler design is discussed. In Section “Modified metaheuristics for the scheduling problem”, modified optimization techniques for smart load scheduling are provided. Section “Results and discussion” discusses the simulation results. Finally, the research is concluded in Section “Conclusion”.

## Architecture of the proposed smart HEMS

**Figure 1:**
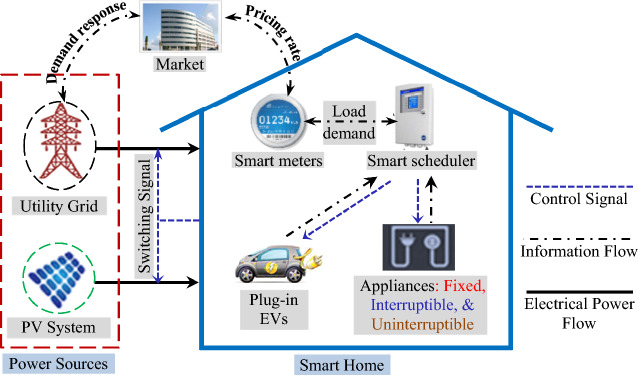
Smart HEMS overall architecture.

As stated previously, smart home technology has been raised as a solution for the traditional power plant issues seeking to reach future smart cities. The consumed energy in the SHs is managed via scheduling the controllable loads according to a smart scheduler built in the HEMS i.e., the designed DSM functions to schedule the connection times (ON–OFF operation) of specific controllable loads that are comprised in the SHs. Commonly, in addition to the smart scheduler, the overall HEMS architecture includes smart meters and smart sensors, as depicted in Fig. 1, which are mostly utilized in monitoring and controlling the energy consumption in SHs depending on collecting energy data, performing energy analysis, and consequently managing the energy usage of different controlled appliances. In addition, plug-in EVs which are considered basic electrical loads of future SHs are considered in this study. The smart meters perform advanced functions such as power quality monitoring and supplying real-time information to utility and users via bi-directional communication i.e., providing the pricing rates and load demand signals transformation between the user and the utility. Sensors embedded in the appliances provide the required information for managing the consumed energy^24^; information flow signals are indicated as dash-dot black lines in the figure. The smart scheduler controls the SHs' electric loads, i.e., the controlled appliances and plug-in EVs, relying on the pricing rate signal, load demand, and customer preferences, the control signals are indicated in the figure as dashed blue lines. The smart scheduler collects ToU tariff signal information as well as power flow to be inputs of the optimization techniques which are designed to get the best solution that meets the scheduling objectives. Depending on the energy-supplying scenario, the energy demand is met by the utility grid (1st powering scenario) or on-grid PV system (2nd powering scenario). The power flow is indicated in Fig. 1 as solid black lines. In the following, more details concerning the modeling of the understudy problem are discussed.

### Residential loads in a typical smart home

The residential electrical loads of a smart home can be classified based on the manner of their operation as schedulable and non-schedulable loads. The non-schedulable loads (fixed loads) are those loads that their operation does not accept interruption or shifting processes i.e., their Length of Operation Time (LOT) and its starting times cannot be changed. In other words, On/Off operation of the fixed loads is done according the as-needed basis, i.e., at the request of the user, which means they have unpredictable operating patterns. Laptops, printers, lights, refrigerator, and hairdryers are examples of these loads. In counterpart, the schedulable loads are controllable loads and have predictable operating patterns. Washing machines, cloth dryer, and water heater, and air conditioners are examples of the schedulable loads. Moreover, in accordance with the impact of supply interruption on tasks, the schedulable loads can be categorized into interruptible (elastic loads) and uninterruptible loads. The interruptible loads are those loads that can be shifted or interrupted during the operating time, such as dish washers, water heaters, washing machines, and clothes dryers. These loads have unidentified but limited LOTs which is determined by the smart scheduler. Whereas, the uninterruptible schedulable loads are those loads that can be delayed or advanced but do not accept to be interrupted during the operating time as they have identified LOTs, such as ovens and fans. Smart schedulers have to schedule these loads between customer's predefined time-slots, accordingly, DSM techniques must be developed to manage complexities of the scheduling process, such as operation time intervals, and to provide the ability to process different types of controllable loads with varying features, such as power consumption, LOTs, and pattern of operation^21^. In this research, the optimal scheduling process includes all the schedulable load types i.e., the interruptible and the uninterruptible appliances in addition to normal charging plug-in EVs, which have been addressed as interruptible electric loads. However, the non-schedulable loads such as fixed appliances and fast charging plug-in EVs, which has been addressed as fixed electric load, have not been considered during the optimization process.

According to the predefined user's needs and constraints, the scheduling process is applied during N-hours time duration, which can be expressed as, $t\in \tau =\{1, 2,\dots \dots \dots , N\}$. The understudy SH is designed to contain diverse sets of loads e.g., a set ${L}_{i}=\{{l}_{i1}, {l}_{i2},\dots \dots \dots , {l}_{in}\}$ of the interruptible loads where $\left|{L}_{i}\right|=n$, a set ${L}_{u}=\{{l}_{u1}, {l}_{u2},\dots \dots \dots , {l}_{um}\}$ of the uninterruptible loads where $\left|{L}_{u}\right|=m$, and a set ${L}_{f}=\{{l}_{f1}, {l}_{f2},\dots \dots \dots , {l}_{fq}\}$ of the fixed loads where $\left|{L}_{f}\right|=q$. Subsequently, the total loads can be grouped in one set $L={L}_{f}\cup {L}_{i}\cup {L}_{u}$ where $\left|L\right|=n+m+q$. Each load $l\in L$ has its own demanded energy for completing its task under predefined operating conditions. For a day-ahead estimated scheduling of the residential loads, the value of $N$ has been taken as 24 i.e., the scheduling process is adjusted as 24-h time duration with hourly sampling. The equations used to model the scheduling problem in this study, i.e., Eqs. (1) to (13), are modified versions of those found in the literature^15,17^. Description of all utilized residential loads and their power ratings, daily usage, and allowed period of operation according to user preferences are given in Table 1. The total power consumed by all the fixed loads, all the interruptible loads, and all the uninterruptable loads at a time-slot $t\in \tau$ (i.e., hourly consumed power) can be expressed by Eqs. (1), (2), and (3), respectively.

1 $${\left.{E}_{fh}\left(t\right)\right|}_{t\in \tau }=\sum_{l\in {{\varvec{L}}}_{{\varvec{f}}}}{E}_{lf}\cdot {S}_{l}\left(t\right)$$

2 $${\left.{{\text{E}}}_{{\text{i}}h}\left({\text{t}}\right)\right|}_{t\in \tau }=\sum_{l\in {{\varvec{L}}}_{{\varvec{i}}}}{E}_{li}\cdot {S}_{l}\left(t\right)$$

3 $${\left.{{\text{E}}}_{{\text{u}}h}\left({\text{t}}\right)\right|}_{t\in \tau }=\sum_{l\in {{\varvec{L}}}_{{\varvec{u}}}}{E}_{lu}\cdot {S}_{l}\left(t\right)$$

where ${E}_{lf}$ represents the power rating of each fixed load $l\in {L}_{f}$, ${E}_{li}$ represents the power rating of each interruptible load $l\in {L}_{i}$, ${E}_{lu}$ represents the power rating of each uninterruptible load $l\in {L}_{u}$, and ${S}_{l}\left(t\right)$ is the ON–OFF status of the load at the time-slot $t\in \tau$, which can be defined by (4),

**Table 1:** Parameters of the household smart loads.

Loads	Power rating (kw)	Daily usage (hrs)	Non-scheduled operating pattern	Operating time window for the scheduling process
Non-schedulable loads				
Lights	1.5	24	All the day	1:00 am:12:00 pm
Refrigerator	1	24	All the day	1:00 am:12:00 pm
Nissan EV	Shown in Fig. 2	1	8:00 am:9:00 am	1:00 am:12:00 pm
Schedulable un-interruptible loads				
Oven	3	3	[1:00 pm:4:00 pm]	12:00 am:8:00 pm
Fan	0.7	6	[1:00 pm:7:00 pm]	1:00 am:12:00 pm
AC	5	11	[2:00 pm:1:00 pm]	1:00 am:8:00 pm
Schedulable interruptible loads				
Washing machine	1	3	[2:00 am:3:00 am, 2:00 pm:4:00 pm]	1:00 am:12:00 pm
Cloth dryer	4	8	[2:00 am:6:00 am, 10:00 am:12:00 am, 10:00 pm:12: pm]	1:00 am:12:00 pm
Water heater	4.5	8	[2:00 am:3:00 am, 5:00 am:6:00 am, 9:00 am:10:00 am, 6:00 pm: 11:00 pm]	1:00 am:12:00 pm
Tesla EV	Shown in Fig. 2	5	[6:00 pm:11:00 pm]	1:00 am:12:00 pm

**Figure 2:**
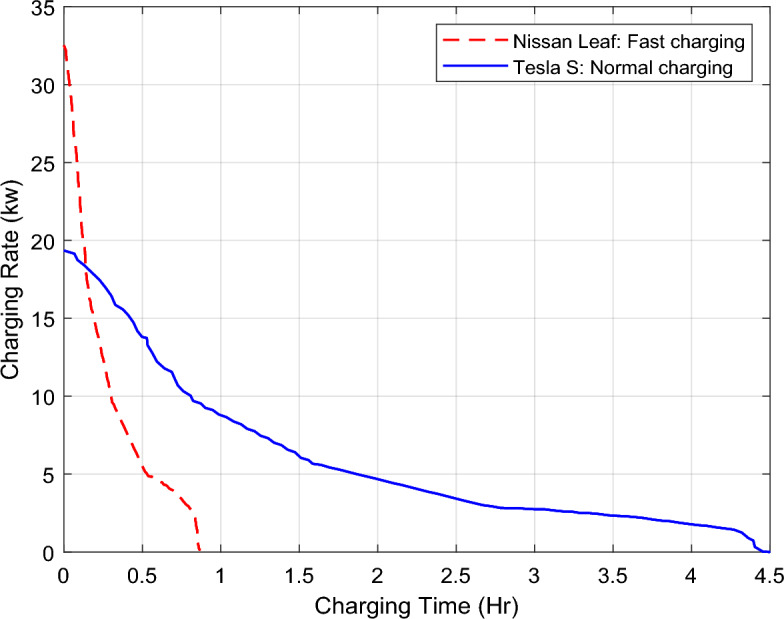
Real-time charging Rate for the understudy electric vehicles.

4 $${S}_{l}(t)=\left\{\begin{array}{c}0\, if\, \mathrm{the\, load\, is\, OFF}\\ 1\, if\, \mathrm{the\, load\, is\, ON}\end{array}\right.$$

The hourly consumed energy by all residential loads ${E}_{TH}\left(t\right)$ can be defined at a time-slot $t\in \tau$ as:

5 $${\left.{E}_{TH}\left(t\right)\right|}_{t\in \tau }={E}_{fh}\left(t\right)+{E}_{ih}\left(t\right)+{E}_{uh}\left(t\right)$$

where the daily consumed energy by all residential loads ${E}_{TD}$ is given as:

6 $${E}_{TD}=\sum_{t=1}^{N}{E}_{TH}\left(t\right)$$

The daily power consumed by all the fixed loads, all the interruptible loads, and all the uninterruptable loads are given by Eqs. (5), (6), and (7), respectively.

7 $${E}_{fD}=\sum_{t=1}^{N}{E}_{fh}\left(t\right)$$

8 $${E}_{iD}=\sum_{t=1}^{N}{E}_{ih}\left(t\right)$$

9 $${E}_{uD}=\sum_{t=1}^{N}{E}_{uh}\left(t\right)$$

### Electric vehicles as future loads of smart homes

Demand for integrating plug-in EVs chargers into power grids becomes essential nowadays as the EVs are expected to be typical loads in power systems in the near future. When an average-use EV is considered, for example a 25 km daily travel range with a 0.1428 kWh/km energy consumption rate, the estimated daily energy consumption is 3.57 kWh. When this EV is charged at 6 kW power, it is found that, one average-use EV is power consumer compared to home electric appliances, e.g., air conditioners and water heaters^25^. Given this, EVs integration into microgrids necessitates proper attention in order to reduce its impact on the power grid stability. For domestic outlet charging and standard charging stations (less than 5 kW on-board chargers), EVs can be charged in a few hours, usually overnight. Recharging EVs with a domestic plug into a regular residential home socket may take 8 to 10 h. Whereas charging at standard station installed at home, EVs may take 4 to 6 h. More powerful stations (5 to 50 kW on-board chargers), called semi-fast charging station, have the ability to reduce the charging time compared to recharging at home, i.e., the full charge range takes around one and half hour. While in order to cut down the charging time greatly, fast charging mode is required in which high-power stations (at least 50 kW off-board chargers) can charge an EV efficiently in under an hour, i.e., from 30 min to an hour, or occasionally a little more based on the charger and the EV^26–28^. In this study, two plug-in EVs have been integrated to the SH, the first one is a Nissan Leaf EV, which has 226 Miles of range, and comes with a 62 kWh Lithium-Ion battery pack. The Nissan Leaf is charged in a fast charging mode (took around 52 min) from 50 to 100% battery State-of-Charge (SOC) and addressed as a fixed load. While the second EV is a Long Range Tesla Model 3, which has 300 miles of range and comes with a 75 kWh Lithium-Ion battery pack. Tesla is charged in a normal charging mode from 10 to 90% SOC and addressed as an interruptible load in this study. The real-time charging rates of these integrated EVs are shown in Fig. 2.

The red line shows Nissan's rate of charge in kilowatts (kW) while the blue line shows Tesla's rate of charge in kW^29^. Daily, the Plug-in Tesla EVs is assumed to arrive at the SH at 6:00 pm, where its charging process is conducted through normal charging mode (for 5 h) during the day, however the charging process of Nissan EV is conducted when requested during the day through a fast charging mode (for 55 min).

### Electricity pricing rates

**Figure 3:**
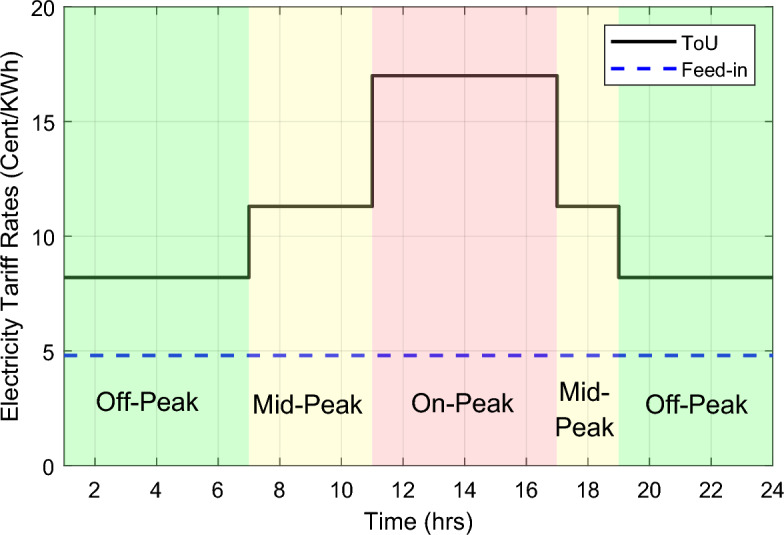
Feed-in, ToU tariff rates.

Different pricing levels are commonly defined during the day by the utility for ToU rates to reflect the saving energy value. High-priced-level tariff is defined for the high-peak hours; this level is known as on-peak tariff. While a pricing level known as off-peak tariff (low-priced-level) is applied during low-demand hours. The former pricing level offers highest electricity cost while the later level offers the lower electricity cost. Intermediate pricing levels may be applied during the day. ON the other hand, feed-in tariff is a policy tool offered by energy retail companies to promote investment in renewable energy sources via encouraging householders to be small-scale producers of PV energy aiming for gaining economic profits. In this study, one summer day pricing has been given as ToU with three tariff levels ranges from the on-peak tariff to the off-peak tariff which have been defined in order as 17.0 cents/kWh, 11.3 cents/kWh, and 8.2 cents/kWh. The pricing levels are shown in Fig. 3 as follows:

The Off-Peak hours are assigned between 7 pm to 7 am at all day weekends and holidays which are indicated in the figure by green shaded areas, the Mid-Peak hours are assigned between 7 to 11 am and 5 pm to 7 pm which is indicated in the figure by yellow shaded areas. Finally the On-Peak hours are assigned between 11 am to 5 pm which is indicated in the figure by red shaded area^30^. Moreover, a flat price is assumed to be 11.0 cents/KWh, whereas a flat rate feed-in tariff is applied regardless of the time of day, it is assumed to be 4.8 cents/kwh.

### Photovoltaic residential system

**Figure 4:**
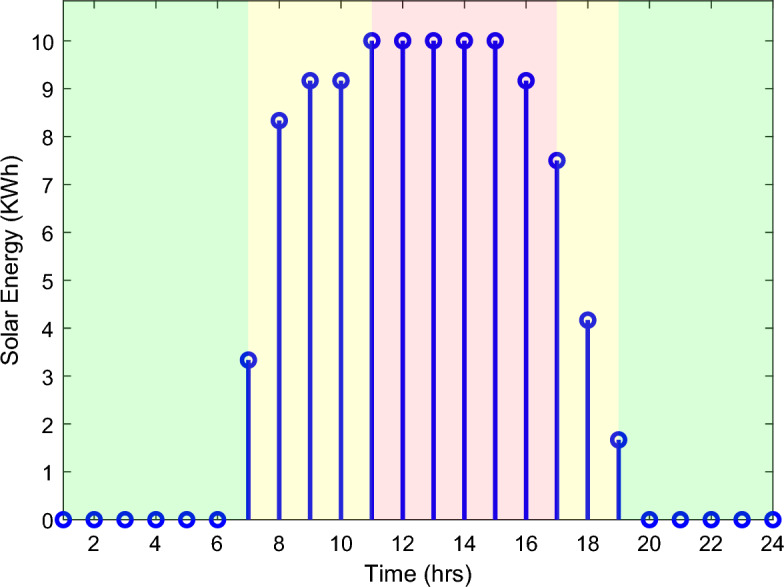
Predicted solar energy.

A photovoltaic residential system of 10.0 KW is installed in the understudy SH, this energy source has no ability to supply the whole amount of electricity demanded by the SH's loads. The solar system is utilized to alleviate the stress on the utility grid especially at high-peak hours (daytimes with highest rates for electricity). The problem of SH's residential loads scheduling has to be solved respecting to the estimated day-ahead PV generation^31^. The estimated solar energy over the day per time unit in a sunny summer day is shown in Fig. 4. It is noticed from the figure that, the generated solar energy covers the on-peak and the mid-peak pricing regions (shown in the figure as red and yellow shaded areas, respectively) indicating that integrating a PV system to the power network positively impacts the electricity bill pricing.

For the designed on-grid PV system, energy flows back-and-forth to and from the utility grid at a time-slot $t\in \tau$ depending on the demand and the solar radiation conditions (i.e., the generated solar energy), simultaneously. The designed HEMS utilizes the best of the power generated by the PV system, thus the consumed solar power ${E}_{cS}(t)$ can be expressed by (10). The generated solar energy may be less or more than the demand ${E}_{T}(t)$ at a time-slot $t$. In the former case, the designed HEMS imports energy ${E}_{cG}(t)$ from the utility grid while in the latter case the HEMS exports the surplus solar energy ${E}_{SS}(t)$ directly to the utility grid and/or sold to another customer in the power network.

10 $${E}_{cS}\left(t\right) = {f}_{min}\left({E}_{S}\left(t\right), {E}_{D}\left(t\right)\right)$$

where ${f}_{min}(a, b)$ is a function for returning the smallest value from two values $a$ and $b$, and ${E}_{S}(t)$ is the total solar generation at time-slot $t\in \tau$. Based on the dynamic pricing signal $\xi \left(t\right)$, the hourly cost ${C}_{TH}\left(t\right)$ of the electricity that is delivered by the utility grid is obtained by (11) whereas the daily cost ${C}_{TD}$ is calculated by (12).

11 $${\left.{C}_{TH}\left(t\right)\right|}_{t\in \tau }={E}_{cG}(t)\cdot \xi \left(t\right)$$

12 $${C}_{TD}=\sum_{t=1}^{N}{C}_{TH}(t)$$

### User comfort

Commonly, householders have the desire that the residential loads complete their tasks promptly within a specified time periods. However, due to some issues in the smart powering systems such as dynamic pricing, high-peak hours, communication delay between the utility and the HEMSs, and loads priority, the customers may endure waiting time for loads' tasks completion. On the other side, there is a trade-off between cost reduction and user comfort as customers give significant concern to cost saving. In order to attain reduced electricity bill while paying attention to user comfort, householders ought to optimally control their controllable loads via utilizing smart scheduler.

Generally, user comfort can be expressed as the waiting time for load tasks completion. In this study, the concept of the waiting time has been considered as the user's waiting time for completing a load task. Basically, there is no waiting time for the fixed loads as their operation must be started and ended directly at user request. The total waiting time ${D}_{T}$ can be formulated as the summation of the delay times of the uninterruptible and interruptible loads. The total delay time of all loads ${D}_{T}$ can be estimated by Eq. (13)

13 $${D}_{T}=\sum_{l\in {({\varvec{L}}}_{{\varvec{u}}}\cup {{\varvec{L}}}_{{\varvec{i}}})}({{t}_{e,sch}-t}_{e,uns})$$

where ${t}_{e,sch}$ and ${t}_{e,uns}$ are the ending times of load tasks in case scheduled and unscheduled scenarios, respectively.

## Formulation of load scheduling problem

As the electricity pricing tariffs are decided by the retail company while the consumer has no ability to modify them, cost saving can be only obtained via scheduling customers' loads in response to the pricing levels during the day i.e., shifting their demand from on-peak hours to off-peak hours. This can be achieved via utilizing smart residential load schedulers which are control programs that provide the ability to run the residential controlled loads on and off during the day for financial incentive and lower electricity bills, while the consumption patterns of the loads are predefined by the user according his preferences. The electricity cost is calculated based on the consumed energy and the dynamic electricity pricing tariff (ToU) which is briefly mentioned in the introduction section. Two powering scenarios for the SH's loads has been nominated in this study. The first scenario is powering from the utility grid only whereas in the second powering scenario an on-grid PV residential system has been utilized.

Commonly, real-world optimization problems include different competing design objectives that cannot be fully achieved as a result^32,33^. In this study, the main objectives of the scheduling problem are simultaneously increasing the saving through reducing the utility bill and keeping the user comfort through reducing the waiting time, besides, a compromise between electricity bill cost and user comfort has been tried. As the multi-objective optimization is challenging programming because of the existence of uncertainties and trade-offs between the problem's objectives, many researches were conducted in the literature on developing multi-objective optimization techniques^23^ and^34^, such as multi-objective particle swarm optimization and multi-objective wind-driven optimization techniques. Searching approaches of those techniques were applied for problems with non-integer decision variables but not for that combine integer and non-integer decision variables i.e., they were designed to find the optimal solutions without considering the integral constraints of the decision variables. The understudy problem has been addressed as a multi-objective constraint mixed-integer optimization problem. The problem objectives are prioritized according to householder needs and constraints through applying the weighted sum strategy, which is a mathematical formulation that has a feature to provide a satisfying solution depending on how an objective is weighted in the overall objective function.

A weighted sum multi-objective function, $J(t)$, of the scheduling problem has been used where boolean variables have been assigned to show whether the loads are ON or OFF. $J(t)$ is expressed mathematically as:

14 $$minimize\, J\left({x}_{i}\right)={w}_{1}\cdot {C}_{TD} + {w}_{2}\cdot {D}_{T}, \forall {x}_{i}\in S$$

where $$S=\left\{{\left.{\left.{D}_{u}\right|}_{l\in {{\varvec{L}}}_{{\varvec{u}}}}\cup S\left(t\right)\right|}_{l\in {{\varvec{L}}}_{{\varvec{i}}}}\right\}$$

subject to:

$${\left.S\left(t\right)\right|}_{l\in {{\varvec{L}}}_{{\varvec{i}}}}=\left\{\begin{array}{c}0\, for\, OFF\, state\\ 1\, for\, is\, ON\, state\end{array}\right.$$

$${\left.{N}_{d}\right|}_{l\in {{\varvec{L}}}_{{\varvec{u}}}}\in \left\{1, 2,\dots \dots \dots , {N}_{max}\right\}, where\, {D}_{u}= {N}_{d}\cdot {T}_{s}\, and\, {N}_{max}\cdot {T}_{s}=24-{T}_{LOT,s}$$

$$\forall l\in {\varvec{L}},\boldsymbol{ }{T}_{LOT,s} \subset {T}_{user}$$

where ${w}_{1}$ and ${w}_{2}$ are two weighting factors, ${T}_{s}$ is the sampling time, ${N}_{max}$ is an integer number, and ${T}_{user}$ is an allowed time period in which the load should complete its task, this period is decided according to user preferences. As the optimization process minimizes $J({x}_{i})$ value, the solution approaching the optima. Whereas the optimization process will be terminated if it is no longer possibility to minimize the bill cost while maximizing the user comfort simultaneously under the prescribed constraints. Normally, the weights are appointed by choosing their values by trial and error in the range from 0 to 1 such that their summation is equal to 1. One of the deficiencies of the weighted sum strategy is that there is no specific method for weights value determination.

As discussed earlier, the utility-decided dynamic pricing and a day-ahead predicted load operating conditions and constraints are utilized to solve the residential load scheduling as an optimization problem over the next 24 h. The objective function of this problem should be minimized subject to linear and integrality constraints on some or all of the decision variables, so that this optimization problem can be considered a discrete optimization problem in which the integrality constraints lets the designed program to capture the discrete nature of the decisions, i.e., the on/off status $S\left(t\right)$ of the interrupted loads at each time step which represent a boolean constraints in the problem. In addition, the delay time of the uninterrupted load ${D}_{u}$ which is integer multiples of the sampling time ${T}_{s}$ is also considered integrality constraints.

## Proposed smart load scheduling design methodology

**Figure 5:**
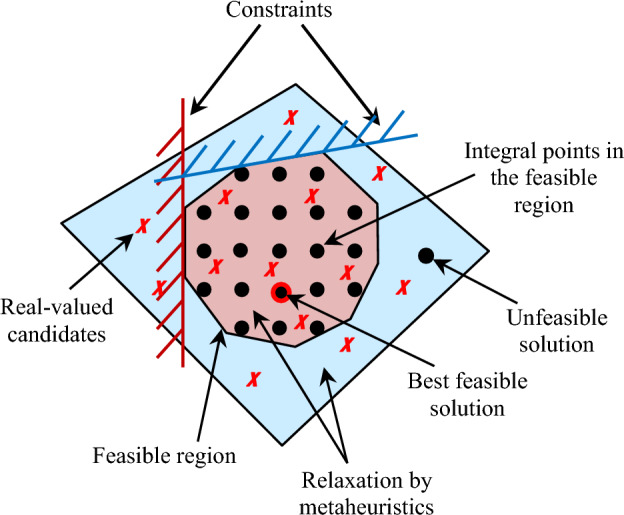
RnR-based non-convex CP-MIP optimization.

Generally, discrete optimization problems can be linear or nonlinear problems, the former comes with linear constraints and linear objective functions while the latter comes with nonlinear constraints and/or nonlinear objective functions. From the formulation and solution algorithms viewpoints, discrete optimization problems can be categorized as pure-integer, mixed-integer, discrete non-integer, and zero–one problems^35^. Thereby, the under study problem can be classified as Mixed-Integer Problem (MIP). In addition, as assignment of the decision variables that satisfy certain constraints is required, this problem can be classified also as Constraint Programming (CP) Problem. Accordingly, the combined MIP and CP (CP-MIP) approach has been utilized to model the scheduling problem i.e., load operation constraints such as interruptible and uninterruptible operations. Generally, CP-MIP optimization problems are non-convex^36^, so that the scheduling problem can be described as nonlinear CP-MIP problem. Relaxation and Rounding (RnR) scheme is commonly applied as a searching scheme to solve such problems for getting the best feasible optimal solution. This algorithm is adopted to map real variables to discrete^37–39^. In this research, modified metaheuristic algorithms have been employed to perform the relaxation process. These algorithms generate candidate solution that may or may not satisfy the integrality constraints i.e., real-valued candidates are generated during updating the current candidates for the next population. Therefore, aiming at obtaining feasible candidate elements that satisfied the integrality constraints to be included in the updated population a developed programming has been proposed through applying the second process in the RnR scheme which is the rounding criterion. The basis of an RnR-based CP-MIP program is shown in Fig. 5. The key insight behind this approach is as follows: at first the under study optimization problem is formulated as a CP-MIP model. Second, relaxation concept is applied to the original CP-MIP problem to have a MIP program which is a linear programing (LP). In the literature, there are two relaxation techniques^40–42^: function-relaxation in which the objective function of the optimization problem is bounded with a function that is easier to deal with such as a convex function, whereas the second relaxation technique is the constraint-relaxation in which the set of feasible solutions is enlarged in such a way that the objective function $J({x}_{i})$ can be efficiently minimized over the enlarged set. In this study, the latter relaxation technique is adopted such that the wider set of decision variables is chosen to be a set of real numbers which can be obtained by eliminating the integrality constraints to let the candidates to take on non-integral values. That is, for each decision variable ${x}_{i}\in S$ where $S=\left\{{\left.{\left.{N}_{d}\right|}_{l\in {{\varvec{L}}}_{{\varvec{u}}}}\cup S\left(t\right)\right|}_{l\in {{\varvec{L}}}_{{\varvec{i}}}}\right\}$, the constraints that restrict the decision variables to be integer are relaxed to let the decision variables to be located in a real-valued region that including the constraints. Third, the resulting LP is solved to allow fractional (rational) optimal solution. To this point, if the obtained decision variables (fractional optimal solution) have integer values, then no more process is required. While if one or more variables have non-integral solutions, then an excessive optimization process (round criterion) is dedicated to those variables in order that their values are more tightly constrained, thus, the solution that satisfies all of the integrality constraints is found. As the feasible domain of the LP is wider than the feasible domain of the CP-MIP, the optimal value of the LP will not be worse than the optimal value of the MIP. So that, the rounded solution is not necessarily optimal for the original problem, however it is not so far from optimal i.e., the rounding algorithm searches for solutions that are good-enough given any other instance^40–42^.

To summarize: in order to get efficient (exact or approximate) solutions for SH's loads scheduling problem, the following strategy has been suggested:

1. Formulate combinatorial optimization problem as CP-MIP to minimize the objective function
  $$\underset{{x}_{i}\in S}{\mathit{minimize\,}}J({x}_{i})$$
  Subject to
  $${x}_{i}\in \{0, 1\}$$
  $${N}_{d}\in \{1, 2, \dots . \dots , {N}_{max}\}$$
2. Derive LP from the CP-MIP by removing the integral constraints, which is called an LP relaxation i.e., relax the constraint ${x}_{i}\in \{0, 1\}$ to ${x}_{i}\ge 0$ and ${N}_{d}\in \{1, 2, \dots . \dots , {N}_{max}\}$ to the inequity ${0\le N}_{d}\le \left({N}_{max}+1\right)$.
3. Continue to minimize the same objective function, but over a (potentially) larger set of solutions.
  $$\underset{{x}_{i}\in {S}_{r}}{\mathit{minimize\,}}J({x}_{i})$$
  Supposing that there is a relaxed set ${S}_{r}$ such that ${S}_{r}$ is a superset of $S$, i.e., $S\subseteq {S}_{r}$, then solve $\underset{{x}_{i}\in {S}_{r}}{{\text{min}}}J({x}_{i})$.
4. Search for optimal LP solution using efficient algorithm. For example, solve the linear relaxation of the CP-MIP efficiently utilizing metaheuristic techniques for linear programming, and let ${x}_{i}^{*}$ denotes an (efficiently selected) optimal solutions over the relaxed set ${S}_{r}$ which are indicated in Fig. 5 by red ${\varvec{X}}$ signs.
  - If the obtained solution has integral values, then it is a solution to the CP-MIP and all is done. Let ${J}_{opt}$ denote the optimal objective function value on the relaxed set; i.e., ${J}_{opt}=J({x}_{i}^{*})$, then the followings are true:
    For each ${x}_{i}\in S$ there is a bound ${J}_{opt}\le J({x}_{i})$.
    If ${x}_{i}^{*}\in S$ then the bound is tighten and ${J}_{opt}= J({x}_{i})$.
  - If the obtained solution has fractional values, then rounding procedure has to be applied to transform the fractional solutions to integral solutions i.e., somehow round the obtained best local solution ${x}_{i}^{*}$ of the relaxed problem to get an approximate solution ${\widetilde{x}}_{i}$ of the original problem which is indicated in Fig. 5 by bold black dots.
  At this step, the approximation algorithm is applied for the understudy CP-MIP problem by employing stochastic rounding process which is a form of rounding procedure that randomly rounds the number up or down to one of the two nearest numbers based on a given probability^43,44^. In this study, the rounding process is implemented to map the obtained best solution ${x}_{i}^{*}\in {S}_{r}$ back to a solution ${\widetilde{x}}_{i}$ that is actually feasible for ${\text{S}}$ depending on a probability criteria with equally chance i.e., rounding the non-integral values to appropriate integers with a switching probability of 0.5. The random rounding, defined by (15), provides the optimization strategy the power to search the best feasible solution randomly at the rounding process^43^. The best feasible solution is represented in Fig. 5 by a hollow red circle. Furthermore, during the optimization process, to force every candidate solution of the optimization process to be allocated with a value different from the value of other candidates in the population, all different constraint concepts ought to be applied. Thus, the assigned decision variables must be an ordering or permutation of the predefined integers.
  15 $${{\widetilde{x}}_{i}=f}_{r}\left({x}_{i}\right)=\left\{\begin{array}{c} \left \lfloor {x}_{i}^{*}\right \rfloor with\, a\, probability\, of\, 0.5 \\ \left \lfloor{x}_{i}^{*}\right \rfloor +1\, with\, a\, probability\, of\, 0.5\end{array}\right.$$
  where $\left \lfloor \cdot \right \rfloor$ is the round of a number toward the negative infinity.

## Modified metaheuristics for the scheduling problem

The proposed RnR scheme has the feature of guarantee getting a feasible best solution in the predescribed domain. In this study, to efficiently find a feasible good-enough solution under the predefined constrains, advanced metaheuristics have been combined with RnR strategy. In the following subsections, four proposed metaheuristic techniques, i.e., the BPSO, SOH-PSO, JAYA, and CL-JAYA, are introduced for the relaxation process. In addition, these techniques are combined with stochastic rounding procedure to search for good-enough feasible solutions. Generally, for all the applied metaheuristics in this study, the optimization algorithms iterate until one of the termination criteria is met i.e., the algorithms examine the termination criteria to terminate the optimization process in case of the values of the global best solution are close enough in some sense for a pre-specified number of iterations, a solution with an appropriate objective function value is obtained, or the maximum number of iterations is attained.

### Binary particle swarm optimization

**Figure 6:**
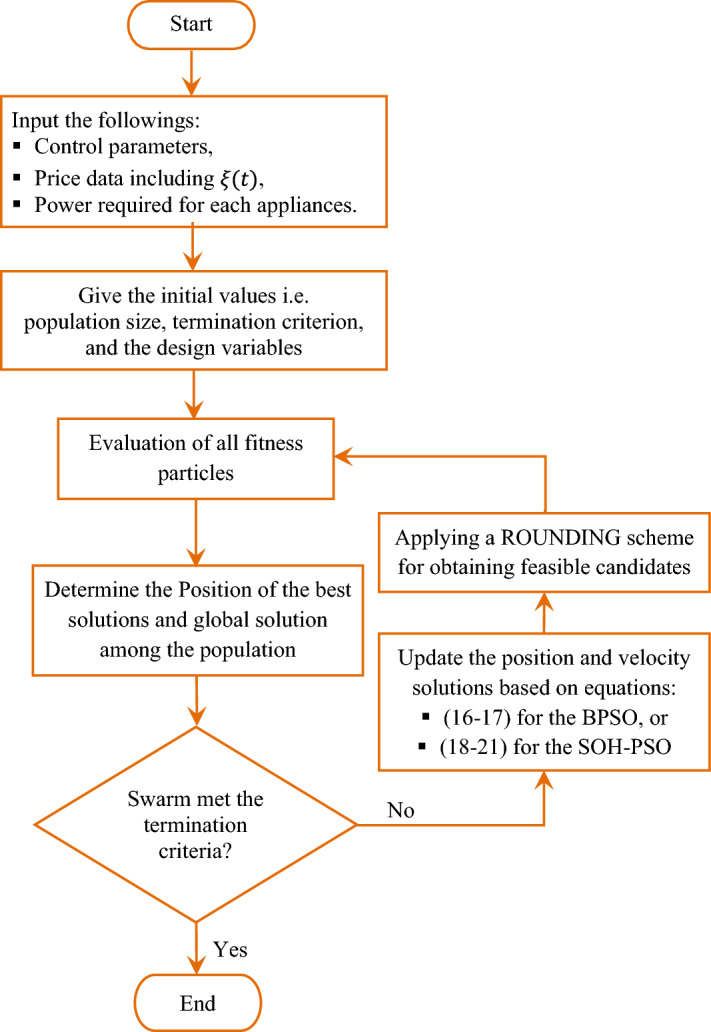
Flow chart for the BPSO and SOH-PSO techniques for RnR-based CP-MIP.

The BPSO is an iterative approach which is introduced by Kennedy and Eberhart^45^. This algorithm is applicable to a very wide range of practical applications. BPSO depends on the concept of population of particles^46–48^. The optimization process is started by allocating initial values to the position and velocities of the particles. BPSO lets the particles (candidate solutions) to group around the optimum solution space to get the best particle (best solution)^49^. Let ${n}_{v}$ is the decision variables number and ${n}_{s}$ particle number. The particles converge toward the optimal solution positions during the optimization process. Each particle is tested during ${n}_{i}$ iterations with the best particle in its neighborhood (local best solution). Accordingly, the best position among local particles at the ${i}{\text{th}}$ iteration is ${{P}_{l}}_{j.k}^{i}$, whereas the global best position is ${{P}_{g}}_{j.k}^{i}$. For $k=\mathrm{1,2},3,\dots ,{n}_{s}$, $j=\mathrm{1,2},3,\dots ,{n}_{v}$, and $i=\mathrm{1,2},3,\dots ,{n}_{i}$, the velocity of the ${k}{\text{th}}$ particle of the ${j}{\text{th}}$ decision variable in the multidimensional search space at the ${i}{\text{th}}$ iteration, ${V}_{j,k}^{i}$, is updated as it moves around the search space as given by (16), whereas the its position vector, ${X}_{j,k}^{i}$, is updated by (17)^48,49^.

16 $${V}_{j,k}^{i+1}=w\cdot {V}_{j,k}^{i}+{c}_{1}\cdot {r}_{1}\cdot \left({{P}_{l}}_{j.k}^{i}-{X}_{j,k}^{i}\right)+{c}_{2}\cdot {r}_{2}\cdot ({{P}_{g}}_{j.k}^{i}-{X}_{j,k}^{i})$$

17 $${X}_{j,k}^{i+1}={X}_{j,k}^{i}+{V}_{j,k}^{i+1}$$

where ${V}_{j,k}^{i+1}$ and ${X}_{j,k}^{i+1}$ represent the updated velocity and position of the particle for the next iteration, respectively, ${r}_{1}$ and ${r}_{2}$ are random numbers between 0 and 1. The two parameters ${c}_{1}$ and ${c}_{2}$ are used to pull the current solution for the local and the global best positions, respectively. The parameter $w$ is the particle's momentum weighting factor. After updating the solutions, the rounding process should be executed to provide feasible solutions under integrality constraints. The accepted feasible solutions obtained by this procedure are employed to update the population for the next generation. The flow chart of BPSO algorithm is given in Fig. 6, for RnR-based CP-MIP optimization.

### Self-organizing hierarchical PSO optimization

The Self-Organizing Hierarchical PSO algorithm is a modified version of the Particle Swarm Optimization technique which has been used in many studies. The SOH-PSO has enhanced solution quality, convergence speed, and escaping capability from trapping in local optima. These features characterize the SOH-PSO technique without decreasing the convergence speed of the best solution^50^. This is obtained in^51,52^, through replacing the global and overall search part obtained from the best value so far $\left({{P}_{g}}_{j.k}^{i}-{X}_{j,k}^{i}\right)$ in (11) with the best non-personal local solution $\left({{P}_{g}}_{j.k}^{i}+{{P}_{r}}_{j.k}^{i}\right)-2{X}_{j,k}^{i}$^53,54^. According to the previous adjustment, searching process utilizing SOH-PSO is expressed by Eqs. (18)–(21) as follows^50^:

18 $${V}_{j,k}^{i+1}={c}_{1}^{i}\cdot {r}_{1,k}\cdot \left({{P}_{l}}_{j.k}^{i}-{X}_{j,k}^{i}\right)+{c}_{2}^{i}\cdot {r}_{2,k}\cdot (({{P}_{g}}_{j.k}^{i}+{{P}_{r}}_{j.k}^{i}) -2{X}_{j,k}^{i})$$

19 $${c}_{1}^{i}={\left|z\right|}^{({z\cdot c}^{i})}$$

20 $${c}_{2}^{i}={\left|1-z\right|}^{(\frac{{c}^{i}}{1-z})}$$

21 $${c}^{i}=\left({c}_{f}-{c}_{i}\right)\cdot \frac{i}{{i}_{max}}+{c}_{i}$$

where $z$ is a standard normal random variable, ${c}^{i}$ is the selecting changing at the ${i}{\text{th}}$ iteration which is selected in a range between the initial changing value ${c}_{i}$ and final changing value ${c}_{f}$ i.e., ${c}^{i}\in \left[{c}_{i} {c}_{f}\right]$. The following step is applying stochastic rounding procedure on the obtained solutions to search solutions in the feasible region. The best candidates during the rounding process are used to update the population for the subsequent generation. The flow chart of SOH-PSO approach is a modified version of BPSO flow chart as illustrated in Fig. 6.

### JAYA optimization technique

**Figure 7:**
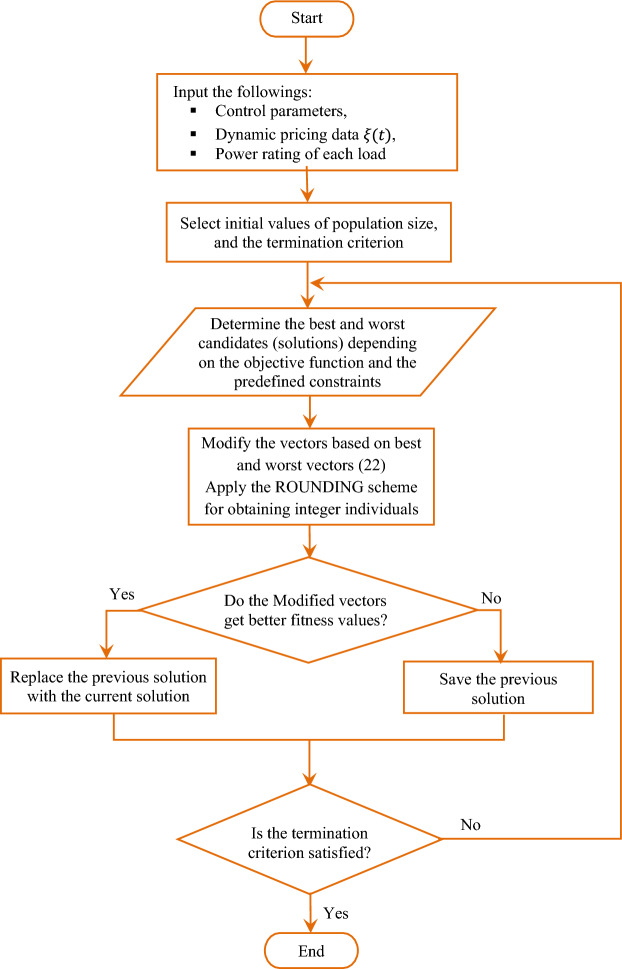
Flow chart of Jaya optimization based on rounding procedure.

One of the main features of JAYA optimization technique is that the optimization process only considers the common control parameters that are the size of the population and the parameters of termination criteria, while the algorithmic-specific parameters are not required. For a problem that has a number ${n}_{v}$ of decision variables and ${n}_{s}$ candidate solutions (population size), the ${k}{\text{th}}$ candidate of the ${j}{\text{th}}$ decision variable, ${\tau }_{j,k,i}$, is updated during the ${i}{\text{th}}$ iteration depending on the integer approximated values of the best and the worst values of the candidates. The updating criteria is defined by the following equation^55,56^:

$${\tau }_{j,k,i}{\prime}={\tau }_{j,k,i}+\left|{r}_{1}\cdot \left({\tau }_{j,b,i}-\left|{\tau }_{j,k,i}\right|\right)-{r}_{2}\cdot \left({\tau }_{j,w,i}-\left|{\tau }_{j,k,i}\right|\right)\right|$$

22 $$\forall k=\mathrm{1,2},3,\dots ,{n}_{s}, j=\mathrm{1,2},3,\dots ,{n}_{v}\, and\, i=\mathrm{1,2},3,\dots ,{n}_{i}$$

where ${\tau }_{j,k,i}^{\mathrm{^{\prime}}}$ represents the updated value of ${\tau }_{j,k,i}$ which is forced to be a positive number via updating the previous solution by a positive value, ${r}_{1}$ and ${r}_{2}$ are two random numbers for the ${j}{\text{th}}$ variable during the ${i}{\text{th}}$ iteration which have values between 0 and 1. The term "${r}_{1}\cdot \left({\tau }_{j,b,i}-\left|{\tau }_{j,k,i}\right|\right)$" expresses the inclination of the solution to be moved toward the best solution, while the term "${r}_{2}\cdot \left({\tau }_{j,w,i}-\left|{\tau }_{j,k,i}\right|\right)$" expresses the tendency to avoid the worst solutions. To this step the updated value of the solution ${\tau }_{j,k,i}^{\mathrm{^{\prime}}}$ has a real value which is only permitted as it achieves a better fitness value. Therefore, for obtaining integer feasible candidates under the integrality constraints, a random rounding procedure is applied on the candidates. The best and worst accepted solutions during the rounding process, i.e., ${\tau }_{j,b,i}$ and ${\tau }_{j,w,i}$, are utilized to update the population for the next generation. The flow chart of JAYA algorithm is given in Fig. 7.

### Comprehensive learning JAYA algorithm

In this research, a modified version of the JAYA technique named comprehensive learning JAYA (CL-JAYA), has been proposed aspiring to improve the global search capability of JAYA algorithm through a comprehensive learning mechanism and application of three learning methodologies: the 1st methodology depends on the current best and worst candidates, same as classical JAYA in (22). The 2nd methodology depends on the mean solution of the current iteration to enhance the chance of JAYA to get-away from trapping into local minimum and to improve searching ability which is achieved with the optimization progress; most of the candidates are grouped around the current best candidate ${\tau }_{j,b,i}$ (performing the local exploitation). The locations of the rest candidates (lagged candidates) are away from the current best candidate (performing the global exploration). During the searching process, at the ${i}{\text{th}}$ iteration, the mean position of the population ${\tau }_{m}$ proceed constantly toward the best solution, as once the optimization process is trapped into a local minimum, ${\tau }_{m}$ guides the lagged candidates allowing higher opportunity to CL-JAYA to get-away from this trap. Taking that into consideration, the 2nd learning methodology depends on the current best candidate and the mean position of the current population to update the solution for the next population as expressed by (23)^57^:

23 $${\tau^{\prime}}_{j,k,i}={\tau }_{j,k,i}+\left|{r}_{3}\cdot \left({\tau }_{j,b,i}-\left|{\tau }_{j,k,i}\right|\right)-{r}_{4}\cdot \left({\tau }_{m}-\left|{\tau }_{j,k,i}\right|\right)\right|$$

where ${r}_{3}$ and ${r}_{4}$ are two random numbers between 0 and 1, and ${\tau }_{m}$ can be defined as:

24 $${\tau }_{m}=\frac{1}{{n}_{s}}\sum_{j=1}^{{n}_{s}}{\tau }_{j}$$

Note that ${r}_{3}$ and ${r}_{4}$ in (23) play the same role with ${r}_{1}$ and ${r}_{2}$ in (22). Furthermore, in the 3rd learning methodology, the current best candidate is considered the guide to speed up CL-JAYA convergence, as expressed by (25):

25 $${\tau^{\prime}}_{j,k,i}={\tau }_{j,k,i}+\left|{r}_{5}\cdot \left({\tau }_{j,b,i}-\left|{\tau }_{j,k,i}\right|\right)-{r}_{6}\cdot \left({\tau }_{j,p,i}-{\tau }_{j,q,i}\right)\right|$$

where ${r}_{5}$ and ${r}_{6}$ are two random numbers between 0 and 1, and $p$ and $q$ are two random integer numbers between $1$ and ${n}_{s}$ whereas $p\ne q\ne i$. The third term in the right-hand side of (25) is used as a random perturbed value to avoid the case when ${\tau }_{j,k,i}$ is the current best solution, that case provides zero value to the second term of (25) leading to get no update for the next population. The previous three learning methodologies are necessary for improving JAYA searching process; so that they should be applied via switching probability criteria with equally chance i.e., same selected probability^57^. Once the best local solution is obtained, a rounding procedure on the obtained best solution is applied to provide an acceptable solution in the feasible domain. The best candidate during the rounding process is appointed to update the population for the next generation. The CL-JAYA algorithm has a flow chart same as shown in Fig. 7 while replacing the learning strategy (22) by the previous proposed switching probability-based learning strategies.

## Results and discussion

**Figure 8:**
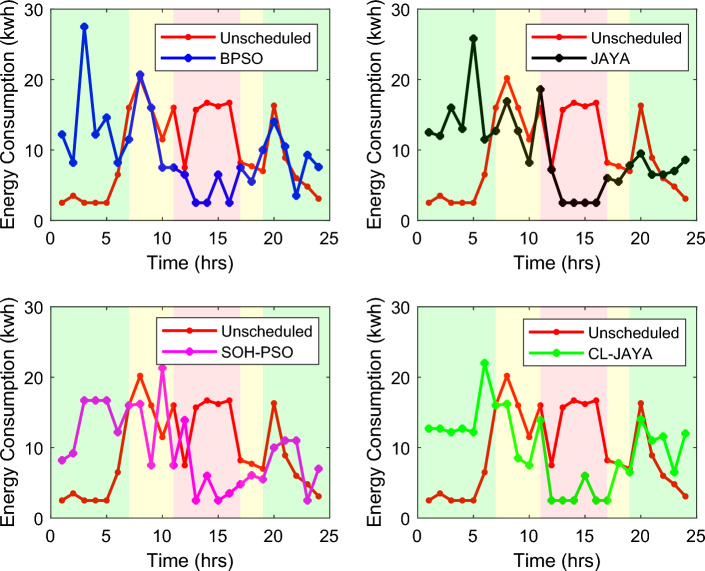
Energy consumption (utility grid only).

**Figure 9:**
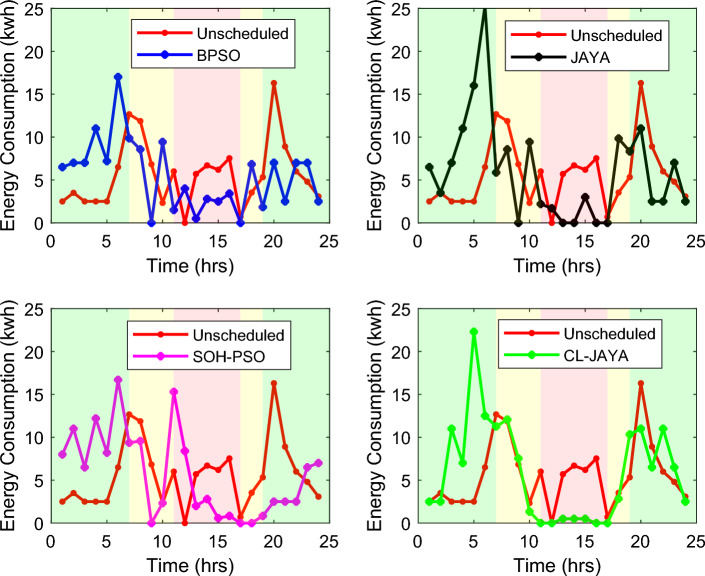
Energy consumption (grid-tied PV system).

**Figure 10:**
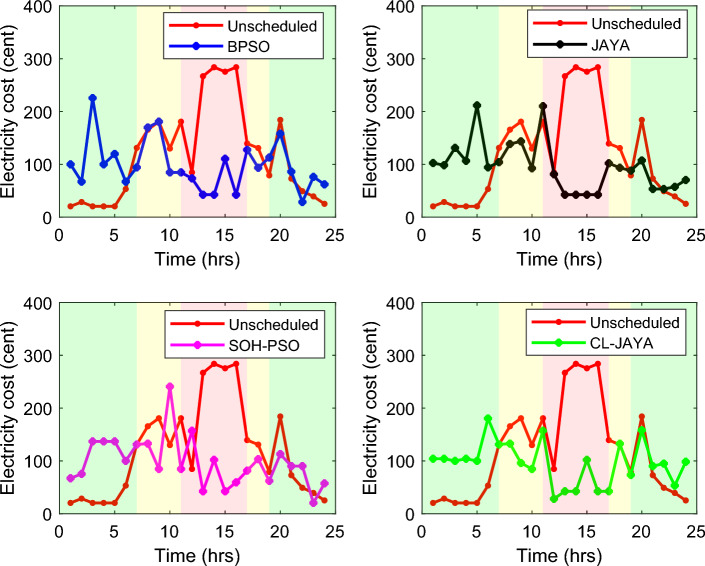
Electricity bill (utility grid only).

**Figure 11:**
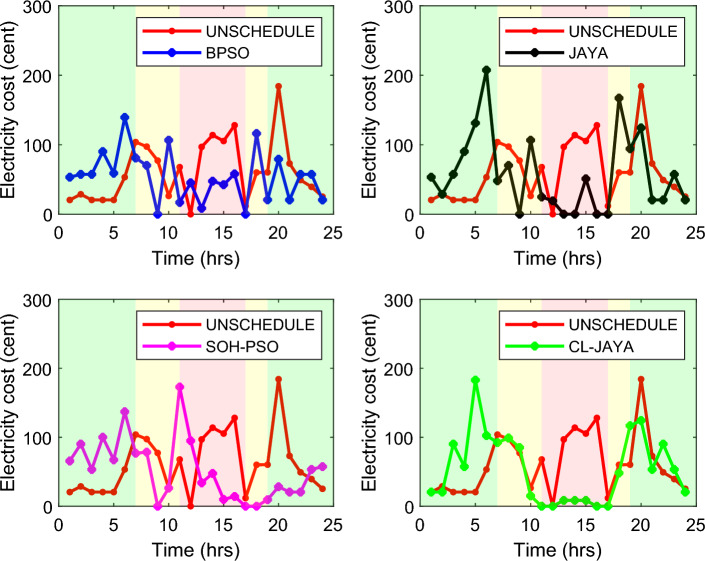
Electricity bill (grid-tied PV system).

**Figure 12:**
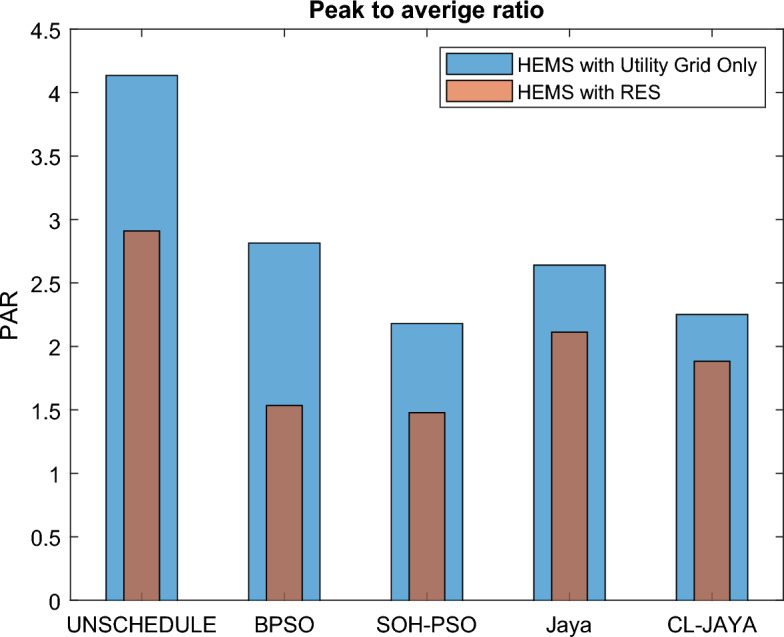
PAR under the scheduled and unscheduled cases of operation.

**Figure 13:**
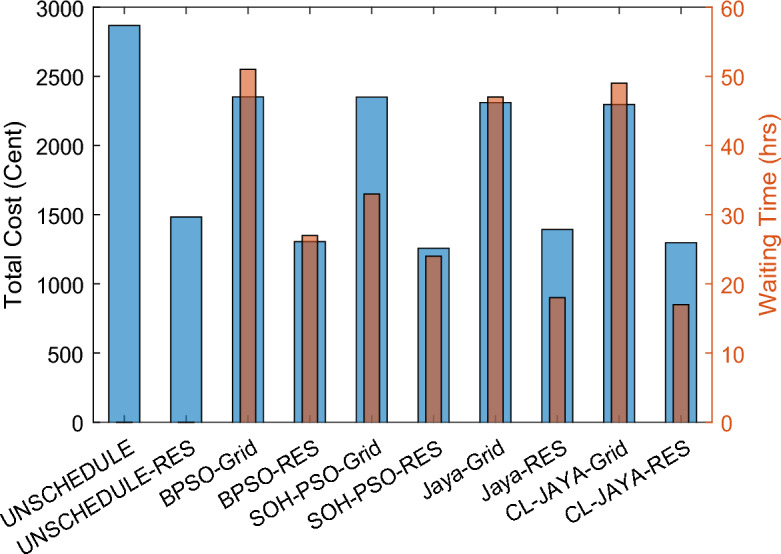
Total cost and overall waiting time.

This section reports the numerical simulation results of the designed smart HEMS which utilizes a modified RnR-based CP-MIP programming that depends on modified metaheuristic algorithms and constraint programming for scheduling the operation of SH's residential loads i.e., the controllable appliances and the plug-in EVs. The simulations have been conducted using MATLAB software to assess the effectiveness of the proposed smart scheduling strategies in improving the utilization of renewable sources, increasing the economic profit and enhancing user comfort. The household is considered under a ToU electricity pricing scheme for residential use, shown in Fig. 3. A model for a smart home has been introduced including eight smart appliances and two integrated plug-in EVs as illustrated in Table 1, where the simulations have been done for 24 h (day-ahead prediction) with an hour scheduling resolution. In this study it is assumed that: the day-ahead PV generated energy in Fig. 4 is accurately predicted. Whereas, the residential loads' power ratings, daily usage, and allowed periods of operation according the user preferences are described in Table 1. Parameters of the applied BPSO, SOH-PSO algorithms have been determined according to^50^, while those of JAYA and CL-JAYA algorithms have been set based on^57^. All of the applied metaheuristic techniques, i.e., BPSO, SOH-PSO, JAYA, and CL-JAYA, show efficient performance in reducing the bill cost as compared to the unscheduled process. Overall, all the proposed techniques provide efficient smart loads scheduling with regard to cost reduction; however, there is a trade-off between cost reductions and user comfort. That is during optimization process, for giving more cost reduction; smart scheduler shifts most of loads other than fixed loads to the off-peak and/or mid-peak pricing regions, thus the waiting time of loads increases which directly impacts the user comfort. The evaluated findings show that the proposed RnR-based CP-MIP programming that depends on the proposed modified versions of PSO and JAYA algorithms outperform the other algorithms in terms of electricity bills cost and user comfort. Finally, in addition to evaluating ToU tariff effectiveness on transferring consumers loads from on-peak hours to off-peak hours, energy exporting tariff (feed-in tariff) shown in Fig. 3, has been evaluated. In the following lines results are shown for two operating scenarios: the first scenario is the operation before implementing the designed DSM whereas the second operating scenario represents the system operation with the DSM implementation. The first scenario is mentioned as "Unscheduled Case", whereas the latter one is mentioned as "Scheduled Case". A comparison has been mentioned regarding the followings: Energy consumption with and without integrating the PV system, electricity bill with and without integrating the PV system, PAR, total cost and overall waiting time. The characteristics of the proposed smart schedulers' performance have been summarized in the Table 2 in terms of daily energy consumption from the grid, total cost, convergence characteristic, Computational efficiency and saving. Whereas, the best fitness value and the number of iteration needed to approach the optimal solution are two criterions used to measure the performance of the different applied metaheuristic algorithms. For the different applied metaheuristics during 100 iterations, the shown convergence characteristics of the best fitness values in Table 2 show that, under the 1st powering scenario, the best fitness value which is obtained by CL-JAYA is better than those of the PSO, SOH-PSO and JAYA algorithms, whereas its computational efficiency is acceptable comparing to that of SOH-PSO algorithm. The PSO algorithm cannot avoid trapping into local optima. Where, under the 2nd powering scenario the best fitness values that obtained by SOH-PSO is better than those of the PSO, JAYA and CL-JAYA algorithms, whereas the SOH-PSO algorithm has fast convergence and its computational efficiency is higher than that of the other algorithms in the comparison. It is concluded that the precision and the efficiency of the modified algorithms are higher than those of the original algorithms. In the following subsections the simulation results are discussed in more details whereas Figs. 8, 9, 10, 11, 12, 13 depict the findings.

**Table 2:** Summary results.

Optimization technique	Powering from the grid only					Powering from the on-grid PV system				
	Unscheduled case	Scheduled case				Unscheduled case	Scheduled case			
		BPSO	SOH-PSO	JAYA	CL-JAYA		BPSO	SOH-PSO	JAYA	CL-JAYA
Daily energy consumption from the grid (Kw)	234.48	234.48	234.48	234.48	234.48	134.46	132.9	135.5	143.7	142.15
Total cost (cent)	2867	2351	2349	2310	2296	1483	1304	1257	1392	1297
Convergence characteristics (best fitness value)	–	1296.3	1249	1270	1243.1	–	706	679.2	699.5	692.5
Convergence characteristics (no. of iterations)	–	3	35	60	53	–	40	48	75	79
Computational efficiency (average time in s)	–	6.74	5.84	6.17	7.82	–	5.94	7.38	5.36	6.32
Saving %**	0	17.9	18	19.4	20	48.2	54.5	56.1	51.4	54.7

**Savings calculation is attributed to the cost in case of powering from the grid only, unscheduled case.

### Energy consumption

Scheduling of the SH's loads for a complete one day (over 24-h time horizon) under the two powering scenarios and utilizing the proposed optimization algorithm is shown in Figs. 8 and 9. Under the 1st powering scenario, the hourly amount of the energy consumed by all loads is provided by the utility, as illustrated in Fig. 8. It is clear from the figure that, comparing to the unscheduled scenario the proposed smart schedulers have the ability to shift the loads to the low-peak and mid-peak pricing regions. In addition, during the non-peak hours (i.e., off-peak and mid-peak hours), the maximum peak of the consumed energy is 20.2 kwh for the unscheduled case, while 21.3 kwh, 22.0 kwh, 25.8 kwh, and 27.5 kwh in case of applying SOH-PSO, CL-JAYA, JAYA, and BPSO, respectively. However, during the on-peak hours, the maximum amount of the consumed energy is 16.7 kwh for the unscheduled case, and 18.6 kwh for JAYA, but it is reduced to 13.9 kwh, 13.9 kwh and 7.5 kwh when applying SOH-PSO, CL-JAYA and BPSO, respectively. Indicating that, the proposed HEMSs (in the scheduling scenarios) successfully shift the load peaks from the on-Peak hours to the off-Peak hours which in turn is reflected on the bill price as will be discussed in the following sub-section.

For the 2nd powering scenario, when a 10.0 KW residential PV system is integrated to the SH's power network, the costumer partially depends on the main grid to power the loads as they are powered from the PV system when available. This scenario provides the designed schedulers ability to flatten the pattern of the demand energy and reduce the peaks of the consumed energy which is reflected directly on the bill price and the PAR and consequently on the grid stability. The energy consumptions of the unscheduled and scheduled loads are indicated in Fig. 9 as hourly patterns (on-grid PV system powering scenario). It is noticed from the figure that, the maximum peak of the consumed energy during the non-peak hours is 16.7 kwh, 17.0 kwh, 22.3 kwh, and 25 kwh when applying SOH-PSO, BPSO, CL-JAYA, and JAYA, respectively, whereas it is 16.3 kwh in the unscheduled case. However for the unscheduled case, during the on-peak hours, the maximum amount of the consumed energy is 7.5 kwh, while it is 15.9 kwh, 3.9 kwh, 2.9 kwh, and 0.5 kwh for SOH-PSO, BPSO, JAYA and CL-JAYA, respectively, implying the effectiveness of renewables to reduce dependency on the utility grid. As summarized in Table 2, the daily energy consumption from the grid in the 1st powering scenario is 234.48 Kw, while it reduces to 134.46 Kw, 132.9 Kw, 135.5 Kw, 143.7 Kw, and 142.15 Kw in the case of the 2nd powering scenario at unscheduled, BPSO, SOH-PSO, JAYA, and CL-JAYA, respectively. Although CL-JAYA accomplishes the highest dependency on the utility grid, but it comes after SOH-PSO in reducing the daily cost of the consumed energy as will be discussed in the next sub-section.

### Electricity cost

Figure 10 illustrates the hourly electricity cost under the 1st powering scenario where the overall demanded energy is provided by the utility grid only. As described in Table 2, the daily cost of the consumed energy in the unscheduled scenario when powering from the grid only is 2867 cents which is decreased to 2351, 2349, 2310, and 2296 Cents while BPSO, SOH-PSO, JAYA and CL-JAYA optimization approaches have been applied respectively. With regard to the total reduction percent of the electricity cost, CL-JAYA technique outperforms the other proposed techniques, as it provides a cost reduction of 20.0%, while BPSO, SOH-PSO, and JAYA accomplish up to 17.9%, 18.0%, and 19.4% respectively. Also, when a 10.0 KWh PV system is integrated to the SH power system, the CL-JAYA offers higher electricity bill reduction comparing to the other proposed algorithms. Figure 11 shows the hourly electricity bill cost with the integrated PV. From the calculations given in Table 2, the daily cost of the electricity bill is reduced to 1570 cents in case of utilizing CL-JAYA, 1475 cents with JAYA, 1610 cents with SOH-PSO and 1563 cents with BPSO. As it is clear from Table 2 and Fig. 11, the cost saving reaches 54.5%, 56.1%, 51.4% and 54.7% in case of BPSO, SOH-PSO, JAYA and CL-JAYA, respectively. These results show that integrating of PV residential system to the powering network able to reduce the electricity bill up to 56.1% daily.

### Peak-to-average ratio

The peak-to-average ratio of a load demand can be defined as the ratio of a user's maximum demand to the average demand during a given time interval, as expressed by (26). PAR provides information that concerns the grid operation and the customers' behavior regarding their energy consumption. Low PAR value improves the grid stability and decreases the electricity cost, while high PAR value weakens grid's stability and reliability and increases the electricity bills^58^. The daily PAR of SH's loads $\Lambda$ can be given as follows, when $N$ is taken as 24 h:

26 $$\Lambda =\frac{{\text{max}}({E}_{T}\left(t\right))}{\sum_{1}^{N}{E}_{T}\left(t\right)/N}$$

Figure 12 shows the performance of the designed smart schedulers for the different utilized metaheuristic techniques on the subject of PAR. It is clear from the figure that, for both powering scenarios, PAR is decreased so far through implementing BPSO, SOH-PSO, JAYA and CL-JAYA comparing to the unscheduled case as the scheduling process flattens the load pattern throughout the day except the on-peak time.

The obtained results for each powering scenario are depicted in Fig. 12. As it is clear from the table, PARs of the unscheduled case are 4.14 and 2.91 for the 1st and the 2nd powering scenarios, respectively; however in the scheduled case, the proposed SOH-PSO technique outperforms all the other techniques significantly as it achieves minimum PARs of 2.18 and 1.48 for the 1st and the 2nd powering scenarios, respectively, whereas, BPSO, JAYA and CL-JAYA provide 2.82, 2.64, and 2.25, respectively for the 1st powering scenario, and 1.54, 2.11, and 1.88, respectively for the 2nd powering scenario. These results indicated that grid stability can be improved with the proposed load scheduling and enhanced more with integrating renewables into the powering network.

### User comfort

As discussed in Sect. 2, user comfort is evaluated based on waiting times for completing loads tasks, so that the worst case occurs when the user experiences long waiting times.. Commonly, user comfort is inversely related to the loads' waiting times and electricity cost. In order to show to which extent the user's convenience has been met by implementing the proposed smart schedulers, the total costs and the overall waiting times of the SH's residential loads under ToU tariffs are shown in Fig. 13. For the 1st powering scenario, comparing to the other techniques BPSO offered longer overall waiting time (i.e., 51 h) while presents the more expensive electricity bill (i.e., 2351 cents), whereas, SOH-PSO allows the most reduced waiting times (i.e., 25 h), while CL-JAYA has minimum cost among the other techniques (i.e., 2296 cents). However, for the 2nd powering scenario, the CL-JAYA presents a minimum overall waiting time (i.e., provides the most enhanced user comfort with 17 h waiting times) while BPSO displays the maximum waiting time of 27 h simultaneously with less saving in electricity bill (i.e., 1304 cents). The obtained results revealed that the objectives of the study can be met with the proposed load scheduling schemes especially with integrating renewables into the powering system.

### Selling solar power back to the utility company

In case of a malfunction existence in the integrated solar system, the designed HEMS depends mainly on utility grid to power the SH otherwise the residential PV system is utilized. However, surplus generation occurs regularly as the produced energy from the PV system and the consumption do not match constantly. Exporting surplus power to the main grid is one of the ways to use surplus power from a solar system. Under the flat feed-in tariffs, customers are credited 4.8 cents per kilowatt hour of electricity exported to the utility via their local solar systems. For the selected day, the solar self-consumption ratio which indicate how much of the electricity produced by the PV residential system has been consumed by the household in case of unscheduled and scheduled scenarios utilizing BPSO, SOH-PSO, JAYA and CL-JAYA are 97.56%, 99.05%, 96.45%, 88.52%, and 90.06%, respectively. Subsequently, the financial gains (daily payback) from the retail company can be calculated as 12.01cents, 4.656 cents, 17.46 cents, 56.46 cents, and 48.93 cents for the same cases, respectively. On the other hand, integrating energy sources from various local producers, such as wind or PV power systems, for serving all of the local users can be considered as a local grid (microgrid), hence the surplus solar energy can be shared with other local energy producers within the local grid. Thus, the local producers have the ability to sell the excess of the produced energy back to peers in the local grid, on a pay-per-use basis, which may provide more financial gains to the customers.

## Conclusion

In this research, a smart load management system has been introduced to schedule daily consumer’s energy usage under ToU pricing tariff. The proposed model provides a day-ahead scheduling solution under two powering scenarios: the first is powering the SH's residential loads from the utility grid only while the second scenario is powering them utilizing an on-grid PV residential system. The objectives of the optimized scheduling problem are reducing the electricity bill and enhancing user comfort. The steps of the proposed technique can be summed up as:

- The Demand Side Management (DSM) problem has been addressed as a multi-objective constraint mixed-integer programming (CP-MIP) optimization problem.
- For enhancing the accuracy and relevance of decision variables, a modified metaheuristics-based Relaxation and Rounding (RnR) approach has been applied as a novel hybrid search technique to solve the understudy non-convex CP-MIP problem.
- The proposed metaheuristics are Binary Particle Swarm Optimization (BPSO), Self-Organizing Hierarchical PSO (SOH-PSO), JAYA algorithm, and Comprehensive Learning JAYA algorithm (CL-JAYA). These algorithms have been applied to relax the CP-MIP to a linear programming to get an optimal rational solution.
- In order to provide a good-enough feasible solution of the scheduling problem, the rounding criterion is carried out via applying a stochastic rounding approach.

Numerical results reported in this research demonstrate that, compared to the unscheduled load case, all the proposed algorithms efficiently reduced the electricity bill. The daily energy consumption from the main grid in the 1st powering scenario is 234.48 Kw, while it reduced to 134.46 Kw, 132.9 Kw, 135.5 Kw, 143.7 Kw, and 142.15 KW in the 2nd powering scenario at unscheduled, BPSO, SOH-PSO, JAYA, and CL-JAYA, respectively. Although JAYA and CL-JAYA accomplish the highest dependency on the utility grid, but they achieved the highest cost reduction of 19.4% and 20.0% respectively, while BPSO and SOH-PSO accomplished 17.9%, and 18.0% respectively. However, SOH-PSO accomplished the highest cost saving of 56.1%, comparing to 51.4% and 54.7% in case of BPSO, JAYA and CL-JAYA, respectively. In addition, the proposed SOH-PSO technique outperforms all the other techniques significantly as it achieves minimum PARs of 2.18 and 1.48 for the 1st and the 2nd powering scenarios, respectively. From the user comfort viewpoint, for the 1st powering scenario, the SOH-PSO allows the most reduced overall waiting time (25 h). However, for the 2nd powering scenario, the CL-JAYA accomplishes the minimum overall waiting time (17 h) i.e., the most enhanced user comfort. The findings of this study are consistent with what was expected when integrating a PV residential system into the SH's powering system, as the electricity bill has been reduced significantly with consideration of the user comfort. Moreover, excessive financial gains are expected in case of energy sharing with other SHs in the local power network.

As a future work, the authors plan to conduct an economic analysis for the practical application of the proposed DSM system and build a prototype for assessing the feasibility of the system as a product for smart home energy management. Further, to evaluate the applicability of proposed scheduling model in a wider range of energy management systems, expanding this research by applying the developed CP-MIP on designing optimal energy management systems for multi-smart home power network with shared photovoltaic and battery storage systems is also targeted a future work.

## Data Availability

All data generated or analyzed during this study are included in this published article.

## Abbreviations

DSM: Demand side management
SHs: Smart homes
PV: Photovoltaic
ToU: Time-of-use dynamic electricity pricing
RnR: Relaxation and rounding approach
HEMS: Home energy management system
BPSO: Binary particle swarm optimization
SOH-PSO: Self-organizing hierarchical PSO
CL-JAYA: Comprehensive learning JAYA
RES: Renewable energy sources
DAP: Day-ahead pricing
CPP: Critical peak pricing
RTP: Real-time pricing
PAR: Peak-to-average ratio
GA: Genetic algorithm
WDO: Wind driven optimization
BFA: Bacterial foraging optimization
EVs: Electric vehicles
LOT: Length of operation time
SOC: State-of-charge
MIP: Mixed-integer problem
CP: Constraint programming
RnR: Relaxation and rounding scheme
LP: Linear programing

## List of symbols

${L}_{i}$*, *${L}_{u}$*,*${L}_{f}$: Set of interruptible, uninterruptible, and fixed loads
$L$: Set of the SH's residential loads
${E}_{fh}\left(t\right)$*,*${E}_{ih}\left(t\right)$*,*${E}_{uh}\left(t\right)$: Hourly power consumed by all the fixed, all the interruptible, and all the uninterruptable loads, respectively
${E}_{lf}\left(t\right)$*,*${E}_{li}\left(t\right)$*,*${E}_{lu}\left(t\right)$: Power rating of each fixed, interruptible, and uninterruptable loads
${E}_{fD}$*,*${E}_{iD}$*,*${E}_{uD}$: Daily consumed power by fixed, interruptible, and uninterruptable loads
${E}_{TH}\left(t\right)$: Hourly energy required by all the SH's loads
${E}_{TD}$: Daily total energy required by all the SH's loads
${S}_{l}\left(t\right)$: ON–OFF state function of SH's loads
${E}_{cG}\left(t\right)$: The imported energy from the utility grid at a time-slot ${\text{t}}$
${E}_{S}\left(t\right)$*, *${E}_{cS}\left(t\right)$*,*${E}_{SS}\left(t\right)$: The total generated solar energy, the consumed solar energy by the SH's loads, the surplus solar energy at a time-slot ${\text{t}}$
${C}_{T}$: The total cost of the electricity supplied by the utility at any time-slot ${\text{t}}$
${C}_{TD}$: The daily cost of the electricity delivered by the utility
$\xi \left(t\right)$: The dynamic pricing signal
${D}_{T}$: The total delay time of all loads
${T}_{e,uns}$: The ending times of load task in case unscheduled scenario
${T}_{e,sch}$: The ending time of load task in case scheduled scenario
${T}_{user}$: The allowed time period in which the load should complete its task
${T}_{s}$: The sampling time
$S$: The feasible region of the decision variables
${S}_{r}$: The relaxed region of the decision variables
$J(t)$: The objective function of the scheduling problem
${x}_{i}^{*}$: Solutions of the relaxed problem
${\widetilde{x}}_{i}$: The approximate solution of the original problem
${w}_{1}$*,*${w}_{2}$: Weight in objective function
${X}_{j,,k}^{i}$*,*${X}_{j,k}^{i+1}$: Position vector of ${k}{\text{th}}$ particle at $i$ and $i+1$ iterations
${V}_{j,,k}^{i}$*,*${V}_{j,k}^{i+1}$: Position vector of ${k}{\text{th}}$ particle at $i$ and $i+1$ iterations
${r}_{1}$: ${r}_{6}$: Random numbers between 0 and 1.
${c}_{1}$*,*${c}_{2}$: Two parameters to pull the current solution for the local and the global best positions
$w$: Weight of the particle's momentum
$z$: Standard normal random variable
${\tau }_{j,k,i}$: The ${k}{\text{th}}$ candidate for the ${j}{\text{th}}$ decision variable during the ${i}{\text{th}}$ iteration
${\tau }_{j,k,i}{\prime}$: The updated value of ${\tau }_{j,k,i}$

## References

[1] United Nations Environment Programme, UNEP. *2021 Global Status Report for Buildings and Construction: Towards a Zero-emission, Efficient and Resilient Buildings and Construction Sector*. (2021).

[2] IEA World Energy Statistics and Balances. https://www.iea.org/. Accessed 5 Jul 2022.

[3] Yi P, Dong X, Iwayemi A, Zhou C, Li S (2013). Real-time opportunistic scheduling for residential demand response. IEEE Trans. Smart Grid.

[4] Quadrennial Technology Review, QTR. *Increasing Efficiency of Building Systems and Technologies* (2015). https://www.energy.gov/sites/prod/files/2017/03/f34/qtr-2015-chapter5.pdf. Accessed 14 Aug 2022.

[5] Smart Home, https://diydivapro.com/5-major-benefits-of-smart-home-technologies/. Accessed 1 Aug 2022.

[6] Javaid N, Ahmed A, Iqbal S, Ashraf M (2018). Day ahead real time pricing and critical peak pricing-based power scheduling for smart homes with different duty cycles. Energies.

[7] Freier J, von Loessl V (2022). Dynamic electricity tariffs designing reasonable pricing schemes for private households. Energy Econ..

[8] Chhualsingh T, Srinivas Rao K, Rajesh PS, Dey B (2023). Effective demand response program addresing carbon constrained economic dispatch problem of a microgrid system. Adv. Electr. Eng. Electron. Energy.

[9] Basak S, Dey B, Bhattacharyya B (2023). Solving environment-constrained economic dispatch for a microgrid system with varying electricity market pricing strategy: A DSM-based approach. IETE Tech. Rev..

[10] Ullah K, Hafeez G, Khan I, Jan S, Javaid N (2021). A multi-objective energy optimization in smart grid with high penetration of renewable energy sources. Appl. Energy.

[11] Zhu Z, Tang J, Lambotharan S, Chin WH, Fan Z (2012). An integer linear programming based optimization for home demand-side management in smart grid. Innov. Smart Grid Technol..

[12] Zhao Z, Lee WC, Shin Y, Song K-B (2013). An optimal power scheduling method for demand response in home energy management system. IEEE Trans. Smart Grid.

[13] Dey B, Basak S, Bhattacharyya B (2023). Demand-side-management-based bi-level intelligent optimal approach for cost-centric energy management of a microgrid system. Arab. J. Sci. Eng..

[14] Ur Rehman A, Wadud Z, Elavarasan RM, Hafeez G, Khan I, Shafiq Z, Alhelou HH (2021). An optimal power usage scheduling in smart grid integrated with renewable energy sources for energy management. IEEE Access.

[15] Hussain HM, Javaid N, Iqbal S, Ul Hasan Q, Aurangzeb K, Alhussein M (2018). An efficient demand side management system with a new optimized home energy management controller in smart grid. Energies.

[16] Liu Y, Xiao L, Yao G, Bu S (2019). Pricing-based demand response for a smart home with various types of household appliances considering customer satisfaction. IEEE Access.

[17] Ahmad A, Khan A, Javaid N, Hussain HM, Abdul W, Almogren A, Alamri A, Niaz IA (2017). An optimized home energy management system with integrated renewable energy and storage resources. Energies.

[18] Nawaz A, Hafeez G, Khan I, Jan KU, Li H, Khan SA, Wadud Z (2020). An intelligent integrated approach for efficient demand side management with forecaster and advanced metering infrastructure frameworks in smart grid. IEEE Access.

[19] Sattarpour T, Nazarpour D, Golshannavaz S (2018). A multi-objective HEM strategy for smart home energy scheduling: Acollaborative approach to support microgrid operation. Sustain. Cities Soc..

[20] Jiang X, Xiao C (2019). Household energy demand management strategy based on operating power by genetic algorithm. IEEE Access.

[21] Abdelraheem S, Abdelhameed EH, Mohamed YS, Diab AZ (2022). Evolutionary techniques-based optimized load management system for smart homes. Int. J. Appl. Energy Syst..

[22] Imran A, Hafeez G, Khan I, Usman M, Shafiq Z, Qazi AB, Khalid A, Thoben K-D (2020). Heuristic-based programable controller for efficient energy management under renewable energy sources and energy storage system in smart grid. IEEE Access.

[23] Hafeez G, Wadud Z, Khan IU, Khan I, Shafiq Z, Usman M, Khan MUA (2020). Efficient energy management of IoT-enabled smart homes under price-based demand response program in smart grid. Sensors.

[24] Moreno Victoria M, Úbeda B, Skarmeta AF, Zamora MA (2014). How can we tackle energy efficiency in IoT based smart buildings?. Sensors.

[25] Ota Y (2018). Electric vehicle integration into power systems. IEEJ Transactions on Power and Energy.

[26] Collin R, Miao Y, Yokochi A, Enjeti P, von Jouanne A (2019). Advanced electric vehicle fast-charging technologies. Energies.

[27] Wolbertus R, van den Hoed R (2020). Fast charging systems for passenger electric vehicles. World Electr. Vehicle J..

[28] Palanisamy, S. & Chenniappan, S. Power quality problems associated with electric vehicle charging infrastructure. in Power Quality in Modern Power Systems, 151–161 (Elsevier Inc., 2021). 10.1016/B978-0-12-823346-7.00005-0.

[29] Charged Electric Vehicles Magazine. https://chargedevs.com. Accessed May 2022.

[30] Hydro Ottawa Holding Inc. https://hydroottawa.com/en/accounts-services/accounts/time-use. Accessed 27 Oct 2022.

[31] Shirazi E, Jadid S (2015). Optimal residential appliance scheduling under dynamic pricing scheme via HEMDAS. Energy Build..

[32] Messac, A. *Optimization in Practice with MATLAB*, 1st edition, 10.1017/CBO9781316271391 (2015).

[33] Anvari-Moghaddam A, Monsef H, Rahimi-Kian A (2015). Optimal smart home energy management considering energy saving and a comfortable lifestyle. IEEE Trans. Smart Grid.

[34] Alzahrani A, Hafeez G, Ali S, Murawwat S, Khan MI, Rehman K, Abed AM (2023). Multi-objective energy optimization with load and distributed energy source scheduling in the smart power grid. Sustainability.

[35] Lindfield G, Penny J (2017). Integer, Constrained and Multi-Objective Optimization. Chapter in Introduction to Nature-Inspired Optimization.

[36] Burer S, Letchford AN (2012). Non-convex mixed-integer nonlinear programming: A survey (2012). Surv. Oper. Res. Manag. Sci..

[37] Umetani S, Yagiura M (2007). Relaxation heuristics for the set covering problem. J. Oper. Res. Soc. Japan.

[38] Anthony Gorry G, Shapiro JF, Wolsey LA (1972). Relaxation methods for pure and mixed integer programming problems. Manage. Sci..

[39] Klotz E, Newman AM (2013). Practical guidelines for solving difficult mixed integer linear programs. Surv. Oper. Res. Manag. Sci..

[40] Helwig S, Hüffner F, Rössling I, Weinard M, Müller-Hannemann M, Schirra S (2010). Selected design issues. Algorithm Engineering. Lecture Notes in Computer Science.

[41] Williamson DP, Shmoys DB (2011). The Design of Approximation Algorithms.

[42] Du D-Z, Pardalos P, Hu X, Wu WL (2022). Relaxation and rounding chapter in introduction to combinatorial optimization. Spring. Optim. Appl..

[43] Croci M, Fasi M, Higham NJ, Mary T, Mikaitis M (2022). Stochastic rounding: implementation, error analysis, & applications. R. Soc. Open Sci..

[44] Connolly, M. P., Higham, N. J. & Mary, T. *Stochastic Rounding and Its Probabilistic Backward Error Analysis, MIMS EPrint 2020.12*, The University of Manchester. 10.1137/20M1334796 (2020).

[45] Kennedy J, Eberhart R (1995). Particle swarm optimization. Proc. IEEE Int. Conf. Neural Netw..

[46] Gudi N, Wang L, Devabhaktuni V, Depuru SSSR (2010). Demand response simulation implementing heuristic optimization for home energy management. N. Am. Power Symp..

[47] Logenthiran, T., Srinivasan, D. & Phyu, E. Particle swarm optimization for demand side management in smart grid. in *2015 IEEE Innovative Smart Grid Technologies-Asia (ISGT ASIA)*, 1–6. 10.1109/ISGT-Asia.2015.7386973 (2015).

[48] Rodriguez M, Arcos-Aviles D, Martinez W (2023). Fuzzy logic-based energy management for isolated microgrid using meta-heuristic optimization algorithms. Appl. Energy.

[49] Abbasi A, Sultan K, Aziz MA, Khan AU, Khalid HA, Guerrero JM, Zafar BA (2021). A novel dynamic appliance clustering scheme in a community home energy management system for improved stability and resiliency of microgrids. IEEE Access.

[50] Ghasemi M, Aghaei J, Hadipour M (2017). New self-organising hierarchical PSO with jumping time-varying acceleration coefficients. Electron. Lett..

[51] Cheng R, Jin Y (2015). A competitive swarm optimizer for large scale optimization. Trans. Cybern..

[52] Liang JJ, Qin AK, Suganthan PN, Baskar S (2006). Comprehensive learning particle swarm optimizer for global optimization of multimodal functions. IEEE Trans. Evol. Comput..

[53] Tehsin S, Rehman S, Saeed MOB, Riaz F, Hassan A, Abbas M, Young R, Alam MS (2017). Self-organizing hierarchical particle swarm optimization of correlation filters for object recognition. IEEE Access.

[54] Ratnaweera A, Halgamuge SK, Watson HC (2004). Self-organizing hierarchical particle swarm optimizer with time-varying acceleration coefficients. IEEE Trans. Evol. Comput..

[55] Venkata Rao R (2016). JAYA: A simple and new optimization algorithm for solving constrained and unconstrained optimization problems. Int. J. Ind. Eng. Comput..

[56] Khan A, Javaid N (2020). JAYA learning-based optimization for optimal sizing of stand-alone photovoltaic, wind turbine, & battery systems. Engineering.

[57] Zhang Y, Jin Z (2022). Comprehensive learning Jaya algorithm for engineering design optimization problems. J. Intell. Manuf..

[58] Kazmi S, Javaid N, Mughal MJ, Akbar M, Ahmed SH, Alrajeh N (2017). Towards optimization of metaheuristic algorithms for IoT enabled smart homes targeting balanced demand and supply of energy. IEEE Access.

